# Less Is More: Oligomer Extraction and Hydrothermal Annealing Increase PDMS Adhesion Forces for Materials Studies and for Biology-Focused Microfluidic Applications

**DOI:** 10.3390/mi14010214

**Published:** 2023-01-14

**Authors:** Larry J. Millet, Anika Jain, Martha U. Gillette

**Affiliations:** 1 Department of Cell and Developmental Biology, University of Illinois at Urbana-Champaign, Urbana, IL 61801, USA; 2 The Micro and Nanotechnology Laboratory, University of Illinois at Urbana-Champaign, Urbana, IL 61801, USA; 3 Biosciences Division, Oak Ridge National Laboratory, One Bethel Valley Road, Oak Ridge, TN 37831, USA; 4 The Center for Environmental Biotechnology, University of Tennessee Knoxville, Knoxville, TN 37996, USA; 5 Neuroscience Program, Beckman Institute for Advanced Science & Technology, University of Illinois at Urbana-Champaign, Urbana, IL 61801, USA

**Keywords:** PDMS, oligomer, solvent extraction, microfluidics, hydrothermal, annealing, cell signaling, astrocyte, glia, imaging

## Abstract

Cues in the micro-environment are key determinants in the emergence of complex cellular morphologies and functions. Primary among these is the presence of neighboring cells that form networks. For high-resolution analysis, it is crucial to develop micro-environments that permit exquisite control of network formation. This is especially true in cell science, tissue engineering, and clinical biology. We introduce a new approach for assembling polydimethylsiloxane (PDMS)-based microfluidic environments that enhances cell network formation and analyses. We report that the combined processes of PDMS solvent-extraction and hydrothermal annealing create unique conditions that produce high-strength bonds between solvent-extracted PDMS (E-PDMS) and glass—properties not associated with conventional PDMS. Extraction followed by hydrothermal annealing removes unbound oligomers, promotes polymer cross-linking, facilitates covalent bond formation with glass, and retains the highest biocompatibility. Herein, our extraction protocol accelerates oligomer removal from 5 to 2 days. Resulting microfluidic platforms are uniquely suited for cell-network studies owing to high adhesion forces, effectively corralling cellular extensions and eliminating harmful oligomers. We demonstrate the simple, simultaneous actuation of multiple microfluidic domains for invoking ATP- and glutamate-induced Ca^2+^ signaling in glial-cell networks. These E-PDMS modifications and flow manipulations further enable microfluidic technologies for cell-signaling and network studies as well as novel applications.

## 1. Introduction

We present a new method for adhering highly biocompatible solvent-extracted PDMS (E-PDMS) microfluidic channels to glass substrates that achieves high-strength bonds without compromising E-PDMS biocompatibility. This approach overcomes limitations of biocompatibility and access to plasma generators that are customarily required for assembling microfluidic platforms, thus enabling labs without plasma generators to fully assemble microfluidics. We overcame this limitation by parameterizing annealing time, temperature, hydrothermal annealing, and glass treatment for native and oligomer-extracted PDMS.

Microfluidic devices are a mainstay for the on-chip miniaturization of chemical and biological systems owing to the highly controlled spatiotemporal manipulations of minute sample volumes [1,2,3]. Microfluidics are particularly beneficial for resolving mechanisms of the growth, differentiation, and signaling of biological systems that are complex and dynamic [4]. Such microtechnology confers the ability to perform complex environmental manipulations that mimic natural systems on a fundamental level to improve the discovery and analysis of biological systems [5,6]. From manipulations of subcellular domains to miniaturized organs-on-a-chip, microfluidics facilitates the systematic interrogations of cells and networks through the establishment of functional microenvironments [5,7,8,9,10,11].

The selection and utilization of materials and chemicals are key factors for defining the cellular microenvironment. Primary and stem-cell derived neurons are sensitive to culture [5]; over the past century, the means of culturing cells of the nervous system have improved in parallel with the development of a range of platforms and media formulations [6]. Recent improvements in microfabrication, materials engineering, and chemical processes have provided organs-on-a-chip that refine the ability to achieve biological discoveries and advance medical diagnostics [7,12,13,14,15,16,17].

Because of the many advantages inherent in PDMS (transparency, affordability, gas permeability, replication, etc.) [18,19], it is widely used for prototyping microfluidic devices. However, the biological implications of PDMS are process-dependent with conditional biocompatibility that scales with device dimensions and possesses fluidic constraints [20,21,22,23,24].

Our previous work demonstrates that PDMS can be rendered highly biocompatible, over native unprocessed PDMS or autoclaved PDMS, by extracting the unpolymerized oligomers and metal catalysts from the cured elastomer to produce E-PDMS [22]. Not only does the solvent extraction process improve the material biocompatibility, but it also alters the material properties of PDMS. E-PDMS exhibits reduced the adhesion of conformal contact to planar surfaces (e.g., glass coverslips, microscope slides, and Petri dishes) [25] and increased the absorption of small molecules [26]. While reduced conformal adhesion of E-PDMS confers benefits for patterning glass surfaces, it also minimizes the transfer of hydrophobic oligomers to the substrate [25]. E-PDMS provides unique material properties; these include fabrication challenges and learning opportunities in material science. An area of study that is lacking focus is the adhesive properties of E-PDMS and annealing protocols to take advantage of these altered properties.

Typical approaches used to covalently bond PDMS to glass substrates include high-energy processes (plasma exposure, corona discharge, UV–ozone exposure) [27,28] that have the potential to break bonds and generate more oligomers. The biological implications of oligomer regeneration in E-PDMS need to be understood more. The presence of oligomers in conventional PDMS microfluidic devices has been shown to influence gene expression [24]; even within ethanol-washed PDMS, oligomers still accumulate within cells cultured in the microfluidic channel [20,24]. Improving highly biocompatible E-PDMS adhesion through non-destructive, bio-compliant processes provides favorable conditions for cell signaling studies in microfluidics and minimizes material-mediated confounds to biological investigations.

We observed in our previous unpublished studies with E-PDMS that cellular processes probe under the E-PDMS to navigate out of the channel between the E-PDMS and glass, indicating a weak and imperfect seal. We also observed distinct increases in adhesion forces for E-PDMS microfluidics in conformal contact after long-term cultures (>10 days), with some E-PDMS microfluidic cultures exhibiting such high affinity for the glass that they could not be removed. Thus, E-PDMS channels in culture either delaminated from the coverslip, or they became increasingly adherent the longer they were maintained in the cell culture environment (a humidified atmosphere at 37 °C). In our unpublished work, we also discovered that by performing solvent-extraction prior to autoclaving, E-PDMS becomes covalently bound to glass substrates. This process also retains the high biocompatibility of PDMS while sterilizing the materials. 

In the first part of this work, we measured and compared the adhesion forces of E-PDMS and PDMS annealed to glass cleaned with two common cleaning methods to resolve a reliable approach for cell signaling studies in E-PDMS. We measure the adhesion forces of E-PDMS as compared to native PDMS over varying regimes of time, temperature, hydrothermal annealing, and glass treatment. We find that, apart from plasma treatment that has the undesirable effect of oligomer generation, autoclaving E-PDMS onto acid-cleaned glass produces the strongest bonds.

**Figure 1:**
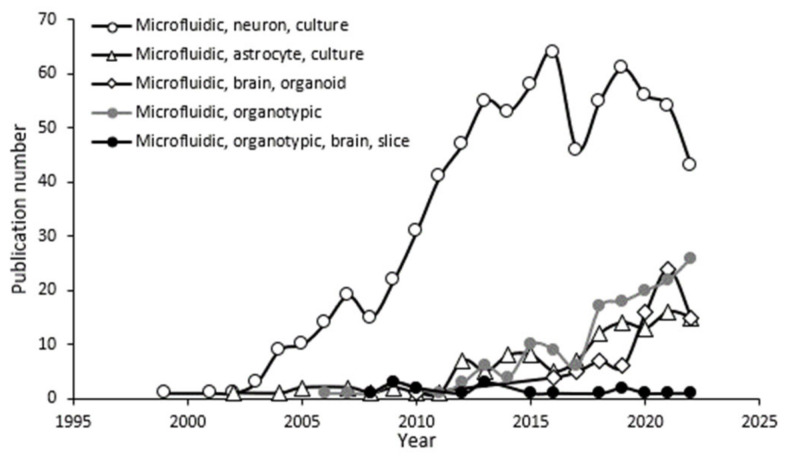
Number of publications for microfluidic preparations of the nervous system categorized by comma-separated search terms defined in the legend. This data was obtained from PubMed search terms (November 2022) for the respective areas of research, e.g., “Microfluidic, neuron, culture”.

In the remainder of this work, we apply these new fabrication techniques to study glial cells, important modulators of brain function [29,30]. These studies are needed for building and studying the organ-level brain function [31,32] of brain organoids in microfluidics [33]. Where neuronal studies have dominated the microfluidic literature landscape over the past 20 years (Figure 1), we focus on interrogating glial network activity by monitoring Ca^2+^ oscillations in response to focal pulses of transmitters applied through laminar flow. Our work demonstrates the advantages of implementing E-PDMS microfluidic platforms for cell signaling applications.

## 2. Materials and Methods

Preparation of PDMS plugs: PDMS plugs were prepared by mixing prepolymer and curing compound (Sylgard 184, Dow Corning) at a 10:1 ratio, respectively. After thorough mixing, all wells of a clean 96-well plate were filled with the PDMS mixture and cured at 70 °C for at least 2 h. Care was taken to prevent filling voids between wells with PDMS. A small amount of 70% ethanol in deionized water (DI, from a Millipore (MilliQ) filtration system) was used to facilitate the release of cured PDMS plugs from individual wells. PDMS plugs were dried of ethanol, then a 1.0 mm dermal biopsy punch was used to bore a diametric hole through the vertical midpoint of the plug. PDMS plugs were separated into two groups for treatment (solvent extraction) and control group (no treatment) prior to assembling plugs onto microscope slides or PDMS slabs.

Preparation of Microfluidic Channels: Microfluidic channels were replicated using the same master sets from our previous work for patterning cell guidance cues on glass surfaces; for master fabrication and PDMS microchannel replication from a library of devices, we refer the reader to our initial description [25]. The cured PDMS microfluidic channel replicates were solvent-extracted through a simplified process (PDMS extraction protocol defined below) followed by a thermal bonding process using an autoclave.

PDMS Extraction: Prior to assembling PDMS plugs or channels onto slides or coverslips, E-PDMS structures were extracted through a series of organic solvents according to the optimized extraction protocol modified from our original process [22], while the control group was left untreated. For microfluidic channels, four to five PDMS microchannel replicates measuring ~464 mm^2^ and 2–4 mm thick were gently stirred and submerged in 150–200 mL of each solvent for the indicated times: HPLC-grade pentane (Fisher Scientific) for ~16 h; xylene isomers with ethylbenzene 98.5+% (xylenes) (Sigma) for 1–2 h; xylenes for 2–4 h; 200-proof ethanol (EtOH) USP for 1–2 h (AAPER); EtOH for 2 h minimum.

In this extraction protocol, pentane is used to swell the PDMS; xylenes are used to remove the pentane; ethanol is used to remove the xylene; water removes ethanol. This gradual solvent exchange returns the PDMS to its former size. In the final step, the PDMS channels were transferred from ethanol to 1 L of sterile DI water and soaked overnight, then dried prior to use. For E-PDMS plugs, the same solvent extraction sequence was employed using 500–600 mL of each solvent. To ensure the PDMS plugs were devoid of any residual solvents, plugs were dried at least 1 week (ambient temperature and pressure) after the extraction process, prior to use.

Glass Cleaning: Two common methods of glass cleaning were employed for comparison, ethanol cleaning and acid-bath cleaning. Ethanol-cleaned microscope slides for PDMS adhesion studies were submerged and spaced apart (not stacked) in a beaker of 200-proof ethanol for at least one day. After soaking, the glass slides were removed and placed on end on absorbent paper to remove excess ethanol and allowed to dry. Acid-cleaned microscope slides were prepared by immersing the glass slides in concentrated sulfuric acid (>90% *w*/*w*) overnight, minimum. For convenience, the slides were kept in a histology rack during acid-bath cleaning. Note of caution: Concentrated sulfuric acid is caustic and hygroscopic; leave ample empty vessel volume for fluid expansion. After a minimum of 24 h in sulfuric acid, the histology rack with slides was removed and rinsed with a direct stream of MilliQ DI for 5 min. Clean glass slides were turned on end and dried. For cell culture, acid-cleaned coverslips (Corning, thickness no. 1.5) were prepared following this same cleaning process.

Elastomer Bonding: Immediately after clean glass slides and coverslips were dry, unextracted and E-PDMS plugs, or microchannels, were placed in conformal contact with the glass substrate for thermal bonding using an autoclave. In a comparative study, ultra-violet light (UV) or a dry oven were used for bonding PDMS to glass substrates; high-powered UV with continuous air flow was used to oxidize/activate the PDMS for bonding, followed by a 70 °C annealing process. The PDMS glass assemblies were placed in an aluminum-foil-lined metal pan, covered, sealed with aluminum foil, and autoclaved with the following settings: 121 °C and 110 kPa for 20 min (sterilization step) with a 20 min drying time (81 °C to 91 °C). After thermal bonding, E-PDMS microfluidic channels for cell culture were cooled, removed from the foil in a sterile biosafety hood, transferred to a culture dish, and prepared for cell culture.

**Figure 2:**
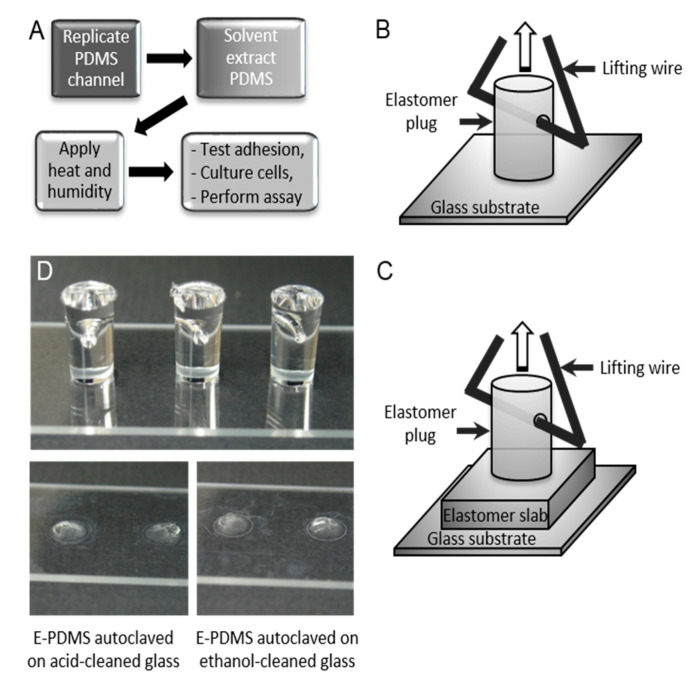
Overview of the annealing process and adhesion force measurements. (**A**) General overview of annealing process, from replicate molding of PDMS through cell culture. For microfluidics, masters are used to generate the PDMS microchannels. For plugs to measure adhesion forces, a 96-well plate serves as the replicate mold. Solvent extraction was performed with n-pentane, xylene, ethanol (200 proof), and DI water. (**B**,**C**) Schematic of PDMS plugs and substrate configurations. Holes punched through the plugs allow for wire supports to attach to the force scales. PDMS on glass (**B**) was used for annealing measures. PDMS on PDMS (“elastomer slab,” (**C**)) was used to measure material deformations through profilometry. (**D**) Images of 3 plugs on a microscope slide for force measurements (upper image). E-PDMS plugs form strong adhesion bonds after solvent extraction and autoclaving. It is typical for E-PDMS annealed after extraction and autoclaving to tear, leaving elastomer fragments when being pulled off the glass, irrespective of glass cleaning method (lower images).

Pull-Off Adhesion Testing: The adhesion testing of adherent unextracted and E-PDMS structures on glass or PDMS substrates was performed at room temperature after autoclaved samples cooled to room temperature (≥30 min). Spring force meters (SI Manufacturing) were used for measuring the normal forces required to separate PDMS plugs from substrates, ‘normal’ here referring to the direction of the force, which is perpendicular to the glass substrate. Meter ranges were in Newtons (N), 0–2.5 N, 0–5 N, and 0–30 N; the footprint of the PDMS plug is 30.7 mm^2^; the results are presented in kPa (1 N/mm^2^). Force meters were connected to the PDMS plugs using a strong wire triangle. The triangle base passed through the PDMS plug with the apex attached to the hook of the force meter (Figure 2B). Force-meter performance was verified with calibrated weight standards. Statistical analyses were performed in GraphPad software (Prism and InStat). Significance was determined (*p* < 0.05) using analysis of variance (ANOVA) testing with repeated measures across time points. *p* values are shown with the conventional “Michelin Guide” scale wherein *p* values for differences are indicated by asterisks *p* ≤ 0.05 (*), *p* ≤ 0.01 (**), *p* ≤ 0.001 (***), *p* ≤ 0.0001 (****).

PDMS Deformation Measurements: Topological measurements of PDMS deformation were performed on a Sloan Dektak3ST Profilometer at the Materials Research Laboratory at the University of Illinois at Urbana-Champaign. Planar pieces of E-PDMS and PDMS were placed on the platform and measured with 2D surface profilometry. Raw data was exported and graphed for comparative analyses.

Flow Control and Manipulation: Dynamic fluid manipulations were achieved by modulating positive and negative pressures at the fluidic ports and reservoirs. Culture perfusion was created with differential hydrostatic pressures and gravity flow. For hydrostatic pressures, fluid droplets were placed at the inlet ports or reservoirs to create positive pressures with surface tension. After surface tensions equilibrated, the dish was tilted a few degrees to elevate one end of the coverslip and reinitiate flow. Negative pressures were obtained with unequal concave menisci at the inlets or reservoir. For pulses of stimuli, rapid fluidic infusion was achieved by combining positive pressures (droplets at inlets) with negative pressures (concave menisci at the outlet reservoir), and the infusate was rapidly reversed by wicking the inlet(s) empty with a Kimtech wipe. The re-initiation of flow was achieved by pipetting defined volumes back into the empty inlet. Imaging controls (with flow of fluorescein and fluorescently labelled antibodies) were performed to refine the pulse process. During glial stimulation, the glial culture channel was always maintained at a greater positive pressure than stimulation channels to prevent stimuli from entering the glial culture compartments. Fluidic manipulations were greatly assisted by monitoring experiment duration during image acquisition and utilizing time-mark features in the Zeiss image-acquisition software.

Glial Cell Culture: Animal procedures were conducted in accordance with PHS Policy on Humane Care and Use of Laboratory Animals under approved protocols established through the University of Illinois at Urbana-Champaign Institutional Animal Care and Use Committee. Astrocytes were isolated from P2-P4 Long–Evans BluGill rats through the following procedure: Subjects were rapidly decapitated; the brain was removed; meninges were cleared from tissues, and the hippocampi and cortex were dissected. For each culture, the tissue was minced and incubated with papain (25.5 U/mL, Worthington) or trypsin EDTA (0.05%) for 30 min at 37 °C. After enzymatic digestion, tissue fragments were rinsed with media (without enzyme), and the tissue triturated through a fire-polished glass Pasteur pipette. The cell suspension was centrifuged at 1400 rpm for 5 min and suspended in astrocyte culture media (DMEM with 10% FBS, 3 mM L-glutamine, and 100 U/mL penicillin and 0.1 mg mL^−1^ streptomycin) and plated at 300–500 cells/mm^2^. Cultures were maintained in astrocyte culture media in a humidified incubator with 5% CO_2_ and 95% air until reaching confluence (~7–9 days). Cultures were shaken at ~300 rpm for four consecutive periods of 18 h to remove unwanted, loosely adherent cells (i.e., microglia); each 18 h interval was interrupted by ~30 h periods of overnight recovery. Astrocytes were released from the dish and loaded into microfluidic channels where they were allowed to grow and extend branches to compartmentalize into adjacent channels.

Calcium Imaging: Imaging spatial dynamics of transient Ca^2+^ signals of individual glial cells and networks was achieved using Fluo-4 AM, a cell-permeable Ca^2+^ indicator dye. Cultures were mounted on a Zeiss LSM-510 Meta NLO laser-scanning microscope without an environmental chamber and imaged at room temperature (22–25 °C). Glial-cell Ca^2+^ transients were evoked with ATP (20 µM) or glutamate (10 µM). Fluorescence signals were obtained using argon laser illumination (488 nm, 0.1%) through a plan-apochromatic 20× (0.8) objective and a LP505 filter. Images were acquired with a photomultiplier tube; detector and scanner parameters (gain, sensitivity, scan rate, zoom and field size) were optimized to minimize laser exposure to live cells and to maximize scan rates for field size and resolution. Profiles of Ca^2+^ transients were aligned (from bottom to top of chart) based on the first occurrence Ca^2+^ transients that affected multiple ROI after stimuli markers; transients of individual ROI may be attributed to spontaneous Ca^2+^ spikes.

Immunocytochemistry: Immunochemistry was performed following our previously published process [22]. Glial cultures were fixed for 30 min with 4% paraformaldehyde, then permeabilized for 10 min with 0.25% Triton in PBS and blocked for 30 min with 10% BSA in PBS at room temperature. To label glial cells, mouse monoclonal primary antibodies against glial fibrillary acidic protein (GFAP, 1:1000 dilution) were used; antibody-labelling occurred for 1–2 h at room temperature or overnight at 4 °C. Secondary antibodies were goat-anti-mouse Alexa 488 (1:1000 dilution, Molecular Probes) incubated at room temperature for 1–2 h. Rhodamine-conjugated phalloidin (5 U/mL, Molecular Probes) was used to label actin filaments (f-actin) with 20 min incubation at room temperature. The same process was followed for immunochemistry in microfluidic channels with flow maintained throughout the labelling process and flow velocities kept to a minimum. Flow directions were reversed every 10 min during the antibody and phalloidin incubations. Following ~20 min PBS rinse, the samples were briefly rinsed with DI water and dried. Channels were filled with Prolong Gold anti-fade reagent (Molecular Probes). To optimize imaging, cells in channels were imaged immediately following antifade reagent application.

## 3. Results and Discussion

### 3.1. Materials Characterization

We hypothesized that longer exposure to elevated temperatures and/or humidity facilitates the formation of adhesion bonds between E-PDMS and a clean glass substrate. To test this hypothesis, we fabricated PDMS plugs of uniform volume and geometry and subjected them to various annealing processes on clean glass slides following solvent extraction. Figure 2 shows the process for measuring the force required to remove each plug from the glass surface to determine bond strengths. The processes of solvent extraction, or autoclaving, alone improves PDMS biocompatibility and renders the microfluidic environment sterile [22], but the combined effects of these processes on adhesion have not been characterized.

**Figure 3:**
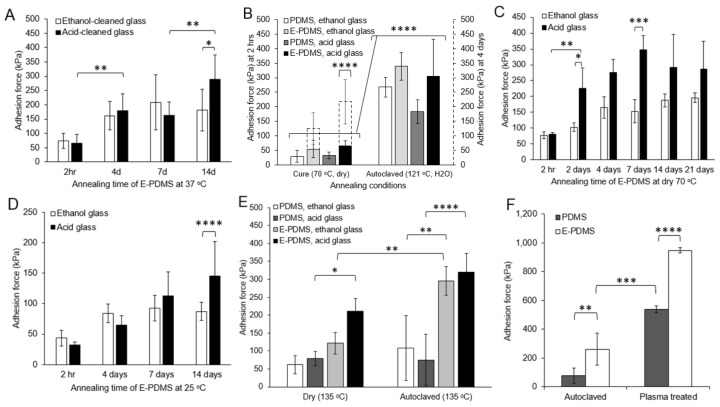
Measurements of removal forces for annealed E-PDMS and PDMS plugs. (**A**) Forces required to remove annealed (humidified 37 °C) E-PDMS plugs off microscope slides were measured. Similar increases in E-PDMS adhesion results are achieved for both ethanol- and acid-cleaned glass at 37 °C. (**B**) Significant temporal effects of two annealing conditions were tested for both PDMS and E-PDMS plugs on ethanol-cleaned glass (white and light grey bars) and acid-cleaned glass (dark grey and black bars) after 2 h processing time. E-PDMS annealed to ethanol- and acid-cleaned glass with dry heat (4 days, dashed bars, secondary *y*-axis) has a significant increase of removal forces over 2 h for E-PDMS on ethanol- and acid-cleaned glass. Autoclave annealing produces superior bonding forces that are statistically significant over dry heat for 2 h and 4 days. (**C**) Temporal-dependence of annealing E-PDMS with 70 °C dry heat measured with greater temporal detail from dashed and solid bars of B. Significant differences are noted between acid- and ethanol-cleaned glass and between 2 h and 2 days and at 14 days. (**D**) E-PDMS plugs kept at ambient temperature (25 °C) and humidity show increased removal forces over a two-week period. (**E**) Measurements of high-temperature-matched annealing (135 °C) under dry or humidified conditions show that heat, humidity, and E-PDMS produce the highest bonding forces. (**F**) Comparison of extraction by autoclave annealing to extraction by plasma-heat annealing. E-PDMS outperforms PDMS in both cases (one-way ANOVA). (**A**–**E**) Two-way ANOVA. *p* values for differences are indicated by asterisks *p* ≤ 0.05 (*), *p* ≤ 0.01 (**), *p* ≤ 0.001 (***), *p* ≤ 0.0001 (****).

Figure 3 shows the increase in adhesion forces of the normal bond plane of E-PDMS plugs on glass slides exposed to a range of conditions (‘normal’ referring to the perpendicular nature of the bonded material). For humid 37 °C cell culture conditions (Figure 3A), there is a substantial increase in the adhesion forces (from 65.1 to 286.6 kPa) of E-PDMS on acid-cleaned glass when exposed for 2 weeks to a humidified atmosphere at physiological temperatures (37 °C). The increase of adhesion forces on ethanol-cleaned glass (Figure 3A, white bars) is more modest and significantly different from the acid-cleaned (black bars) surface at 14 days (n = 8, two-way ANOVA). Our glass cleaning process for primary cell culture uses a sulfuric acid bath followed by a wash with copious amounts of DI water. A common alternative cleaning process is sterilization with ≥70% ethanol in DI water. Our results (Figure 3A) show that, under cell culture conditions, acid-cleaned glass enables a stronger adhesion of E-PDMS to glass than does the ethanol-cleaned glass.

### 3.2. Hydrothermal Annealing

To resolve the contributions of conventional fabrication and sterilization conditions to increased E-PDMS bonding strengths, we compared E-PDMS annealed to glass with dry heat (PDMS curing oven, 70 °C) vs. humidified heat (autoclave sterilization, 121 °C). Figure 3B shows that autoclaving increases the adhesion of E-PDMS and PDMS over dry-heat exposure for both material types and glass cleaning processes (n = 8, two-way ANOVA). E-PDMS showed superior adhesion over PDMS, regardless of treatment. The dashed data bars of the secondary *y*-axis (Figure 3B) show that, after 4 days exposure of E-PDMS and PDMS to dry heat, PDMS adhesion results were not statistically different from 2 h, whereas E-PDMS on acid-cleaned glass over 4 days showed a statistically significant increase (from 65.1 kPa to 218.2 kPa, n = 8, two-way ANOVA); the increased adhesion on ethanol-cleaned glass from 2 h to 4 days was not significant.

We performed an extended time-course study on the temporal-dependence of E-PDMS annealing under dry heat (Figure 3C) to further resolve the influence of dry-heat annealing shown in Figure 3B. Figure 3C data show that, after 7 days at 70 °C, E-PDMS normal adhesion forces (153.1 to 348.5 kPa) are significantly greater (n = 4, two-way ANOVA) on acid-cleaned glass than the alternative, and normal adhesion forces become similar to those achieved through the 2 h autoclaving process shown in Figure 3B. These data suggest that increased heat promotes the annealing of E-PDMS to glass. Figure 3D shows that E-PDMS plugs kept at room temperature (25 °C) for 14 days did not show as large of an increase in normal adhesion forces as dry heat (Figure 3C) but at 14 days, it had significantly stronger adhesion (Figure 3D, 87.2 kPa verses 145.7, n = 8, two-way ANOVA) on acid-cleaned glass than on ethanol-cleaned glass. Figure 3E shows the influence of temperature and humidity on adhesion forces. We exposed both E-PDMS and PDMS plugs to temperature-matched (135 °C, 20 min) treatments in a dry oven and in an autoclave (saturated humidity). Autoclaving produced significantly higher normal adhesion forces (n = 4, two-way ANOVA) than dry heat for E-PDMS on acid-cleaned glass and ethanol-cleaned glass. Again, E-PDMS adhesion outperforms PDMS, and acid-cleaned glass outperforms ethanol-cleaned glass for 135 °C humidified and dry annealing conditions. Autoclaving provides superior results for E-PDMS, suggesting that both humidity and elevated temperatures facilitate the annealing process of E-PDMS to glass. It should also be noted that it is possible for PDMS and E-PDMS structures to have altered dimensional stability and tear strength after autoclave sterilization [34].

For perspective, we compared our annealing process for binding E-PDMS to glass to the typical oxygen plasma-bonding process. E-PDMS material can be activated through high-energy processes; however, these high-energy processes break PDMS bonds and introduce more oligomers into the culture system [28,35,36]. Figure 3F shows that E-PDMS adhesion outperforms PDMS after autoclaving or plasma treatment, and plasma treatment shows superior normal adhesion forces compared to autoclaving. It remains uncertain the quantity of oligomers that are generated in E-PDMS through high-energy processes, such as plasma treatment, or how freely any newly formed E-PDMS oligomers can translocate from the material to cells cultured in microfluidic systems. From these data, the solvent-extraction of unbound oligomers and catalyst from PDMS increase bonding forces through a thermal hydration process.

It is known that siloxane and silanol groups equilibrate with a humidity-dependence; silanols are very unstable, and the reaction is highly skewed in favor of forming thermally stable siloxane bonds through a condensation reaction [37,38,39,40]. Nevertheless, hydrogen bonding, van der Waals forces, and nano-scale interlocking interactions of the glass and PDMS cannot be underestimated and could also contribute to increased adhesion, in addition to siloxane bond formation. Even the inclusion of silica filler particles in PDMS can influence the material properties, thus having further implications for PDMS extraction [41]. Given these adhesion study results, we employed solvent extraction followed by autoclave annealing to begin understanding oligomer translocation in and between PDMS types.

### 3.3. PDMS Deformation and Oligomer Translocation

**Figure 4:**
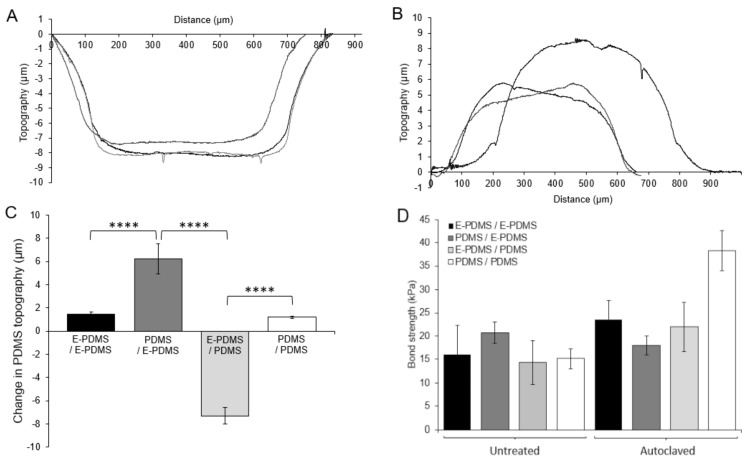
Measurement of deformation and removal forces for E-PDMS and PDMS in direct contact with each other. (**A**,**B**) Stylus profilometry data (Sloan Dektak3ST Profilometer) confirms the visual observations and profiles topological changes of E-PDMS and PDMS deformation when extracted and non-extracted PDMS samples are paired in direct contact and autoclaved. (**C**) Summary of topography changes of PDMS and E-PDMS material slabs from profilometry data of E-PDMS and PDMS plugs processed in pairwise combinations. The PDMS/E-PDMS combinations show much greater changes (3 repeats (n = 3 each), one-way ANOVA) than both E-PDMS/E-PDMS- and PDMS/PDMS-matched material types, which show no significant changes. (**D**) Adhesion testing measurements of untreated and autoclave-annealed E-PDMS and PDMS plugs on E-PDMS and PDMS elastomer slabs. Weak adhesion forces for PDMS on PDMS are higher than any E-PDMS interactions and are attributed to the remaining oligomers and metal catalysts left in the bulk polymer (no significance). *p* values for differences are indicated by asterisks, *p* ≤ 0.0001 (****).

With the ability to covalently bond E-PDMS to glass through an autoclaving process, we investigated the possibility of performing polymer-to-polymer bonding through combinations of native PDMS and PDMS extraction combined with hydrothermal annealing via an autoclave. We placed polymerized E-PDMS and PDMS material plugs directly on planar slabs of E-PDMS and PDMS for all-pairs testing in a combinatorial method. We then subjected the material pairs to the same autoclaving process. Following the autoclave annealing process, we observed a consistent trend of noticeable location-dependent deformation of the plug “footprint” where the material plugs were in contact with the opposite substrates (i.e., E-PDMS on PDMS, PDMS on E-PDMS). A stylus profilometer was used to obtain profile measurements (Figure 4A,B) of material height; the data show that PDMS substrates visibly indent (~7 µm depression) where E-PDMS plugs are placed in direct contact during autoclaving. Conversely, the footprints on E-PDMS substrates swell (~6–9 µm elevation) only where PDMS plugs are in contact with E-PDMS substrates (Figure 4B). The footprint deformation induced by autoclaving E-PDMS in contact with PDMS is significant (Figure 4C, n = 3, two-way ANOVA). The direction of deformation is consistent regardless of whether E-PDMS serves as the plug or substrate (Figure 4C). No appreciable changes of footprint deformation are observed when E-PDMS and PDMS plugs are matched to the self-same material slab (PDMS on PDMS, E-PDMS on E-PDMS).

Oligomer translocation is possible in cross-linked PDMS, as evidenced by the time-dependent reversion of plasma treated PDMS from a hydrophilic to hydrophobic state [35,36,42,43]. This information, combined with our results of E-PDMS and PDMS deformation, suggests that oligomer translocation occurs within PDMS, and the effect on the material is measurable after translocation from oligomer source (PDMS) to oligomer sink (E-PDMS). The observable deformation indicates that a pronounced quantity of material remains unpolymerized when prepared under common conditions (10:1 ratio, 70 °C cure for 2 h). It also provides further evidence to support the need to remove unpolymerized oligomers through the pentane–xylene–ethanol–water solvent extraction process.

Measurements for PDMS adhesion with types of PDMS pairing show that the highest bonding strengths (38.4 kPa) occur for the PDMS–PDMS combination over any of the other pairings of the two polymer types (Figure 4D), possibly due to the presence of oligomers and the platinum catalyst that can facilitate further chemical interactions between untreated PDMS materials at elevated temperatures. Without oligomers and catalyst in one of the two paired materials, bonding strengths are on average half (12.7–25.4 kPa) those achieved with PDMS–PDMS interfaces. Solvent extraction removes oligomers and platinum catalyst from the polymerized material [22], thus reducing its ability to crosslink with other PDMS surfaces but improving the ability of E-PDMS to bind to glass. These results provide new data and perspective that adds further evidence to the understanding that E-PDMS possesses unique material properties, beyond increased biocompatibility, apart from its native, unextracted material form.

### 3.4. Fluidic Manipulation

**Figure 5:**
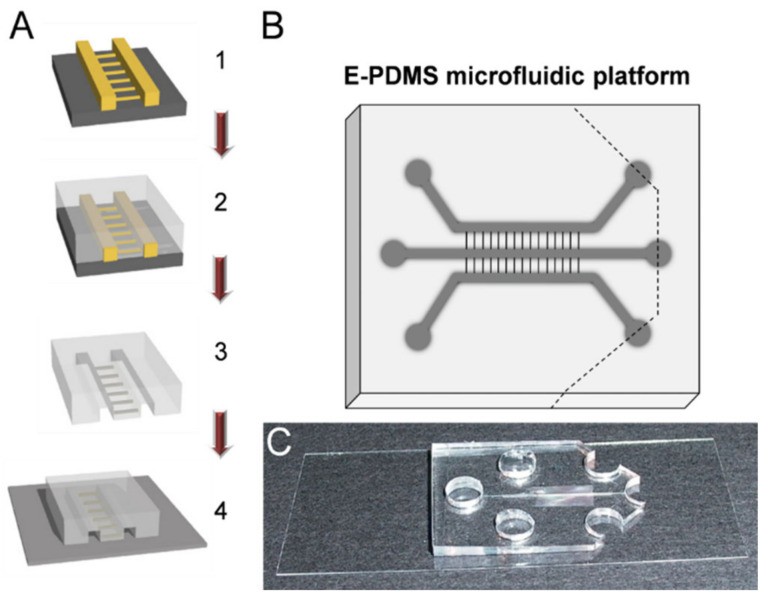
Process flow for fabrication and assembly of microfluidic devices for cell signaling studies. (**A**) Overview of fabrication and assembly process for microfluidic chambers (200 μm W × 45 μm H) for cell signaling. Step 1: Microfluidic masters are used for (step 2) replicate molding of PDMS-based microfluidic channels with interconnecting cross channels. Step 3: PDMS replicates are trimmed, and holes punched (upper right diagram) prior to solvent extraction to remove un-cross-linked oligomers and metal catalysts. Step 4: E-PDMS replicate is annealed to the glass substrate through autoclave sterilization, completing the microfluidic device. (**B**,**C**) Diagram of microfluidic architecture (**B**) and photograph (**C**) of an annealed E-PDMS microfluidic device used for cell signaling studies. Ports are cut with dermal biopsy punches (5–6 mm). The reservoir is made by bisecting three ports on one end of the platform (dashed line).

**Figure 6:**
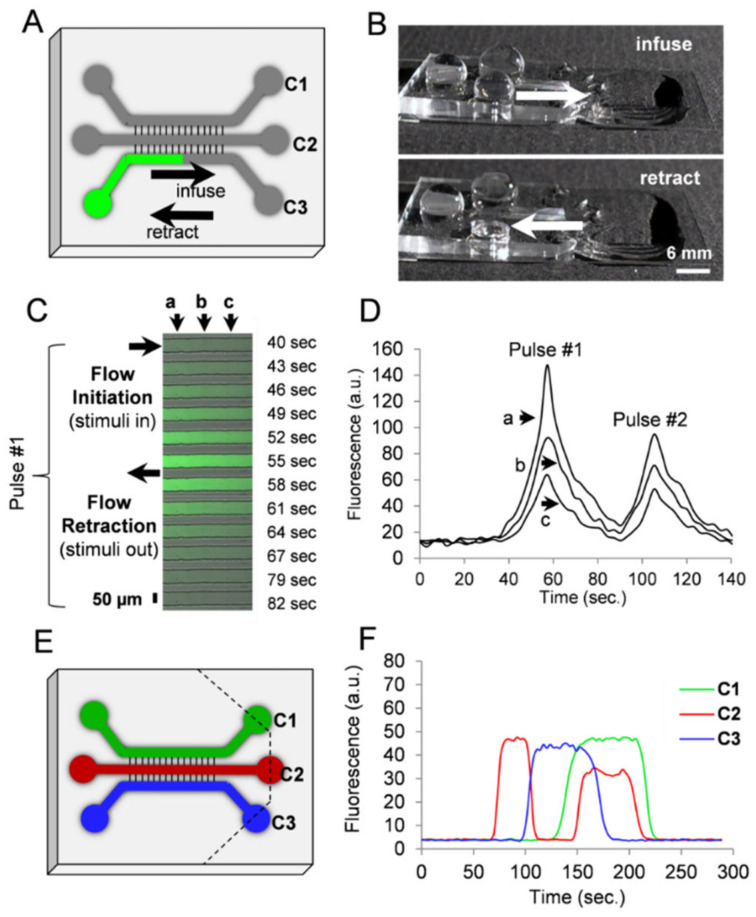
Chemotemporal pulses in microfluidic channels. (**A**) Schematic and image of PDMS channels on a coverslip for a fluidically connected microfluidic platform for cell signaling. (**B**) Using surface tension and gravity flow, chemicals can be pulsed in the channel for stimulating compartmentalized cells. (**C**) For pulse characterization, dilute FITC-conjugated secondary antibodies (green) were introduced in the channel. Spatial characteristics were assessed at three separate points 125 µm apart (a, b, c). Data for panel (**C**) was acquired from the midpoint of channel C3 as shown in the schematic of panel A. (**D**) Two serial pulses demonstrate spatiotemporal characteristics for multiple cellular stimulations. In pulse #1, half-maximal stimulation concentration is achieved at peak (a) with a concentration decay of 40% at peak (b) and 57% at peak (c). Positions of points a, b, c as in (**C**). (**E**) Parallel pulsatile flow actuated by surface tension and gravity-mediated passive pumping within the same device. The device was cut along the dashed lines to allow for unrestricted outflow. Pulsatile flow was accomplished as shown in (**B**) with an increased fluid head at the inlet initiating channel infusion and the emptying of the inlet, resulting in flow retraction. (**F**) Flow in the three individual channels (C1, C2, C3) can be modulated independently to produce spatiotemporal pulses as depicted in the profiles of fluorescence. Flow was initiated in C2, then retracted in C2 at the same time as C3 initiation; infusion of C1 and re-infusion of C2 precede retraction at C3, ending with serial retractions from C2 and C1.

With the ability to produce highly biocompatible E-PDMS tightly bound to clean glass substrates, we created microfluidic platforms to enable thr spatiotemporal manipulation of fluids to achieve multiple, simultaneous or sequential focal stimulations across compartmentalized microfluidics. Following solvent extraction, E-PDMS microfluidics were trimmed to produce fluid source wells useful for retaining and perfusing media during cell culture, for actuating fluids during cell signaling studies, and for immunolabelling cells. Figure 5 shows an overview of the stepwise microfluidic assembly process used here. Fluidic pulses, achieved through hydrostatic pressure, were produced in adjacent channels of the microfluidics to demonstrate a range of fluidic controls from seconds to minutes (Figure 6). This method of chemo-temporal manipulation shown in Figure 6A,B enables rapid chemical transients without pumps increasing pressures within the microfluidic device. Figure 6C shows a time-lapse image series for a short segment (350 µm) of channel 3, as shown in Figure 6A. Figure 6D shows the changes in the fluorescent intensity of FITC-conjugated antibodies pulsed into the channel by an injection–retraction technique (Figure 6C). Figure 6E depicts the same chip architecture used for multiple fluidic pulses (Figure 6F) in a single timeframe.

### 3.5. Cell Signaling in Microfluidics

**Figure 7:**
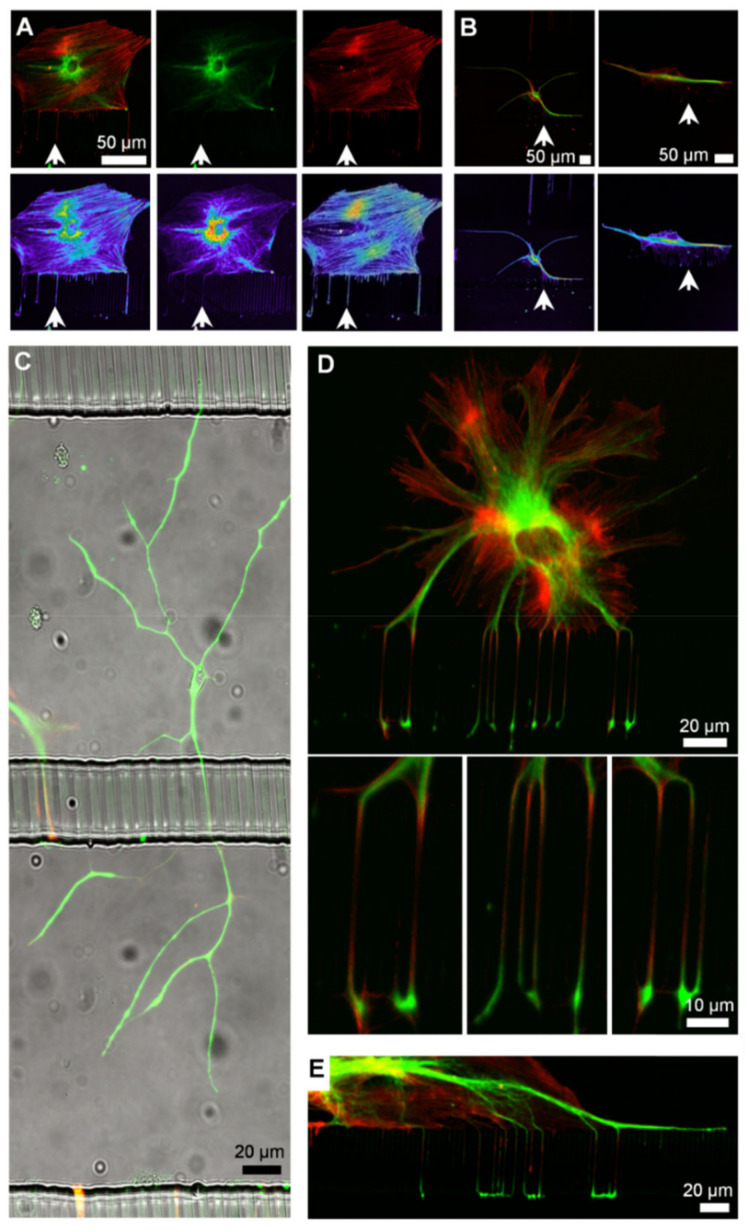
Compartmentalization of glia in microfluidic channels. (**A**,**B**) Glial cells cultured in contact with E-PDMS microfluidics develop cellular branches that extend through interconnects. Antibodies for glial fibrillary acidic protein (green) and rhodamine phallotoxins (phalloidin, red) label the cytoskeleton. Fluorescence intensities of cell branches are represented by glow-scale intensity images (below). Fine, filamentous glial branches are rich in filamentous actin and contain GFAP filaments. Filamentous cellular protrusions of glia extend through the microfluidic channels (white arrow). (**B**) Merged fluorescence images of two glial cells are shown, each in its own image pane. (**C**) Large (~400 μm long), ramified glial cells do not show affinity for channel sidewalls and interconnects as most glial cells do. (**D**) A glial cell in the top channel extends branches into the bottom channel allowing subcellular stimulation with signaling molecules applied through adjacent channels. Insets show magnified views of glial branches wrapping around the interconnect pillars, merging as they emerge into the bottom channel. (**E**) Glial cells often spread along the interconnect wall sending branches over distances exceeding 100 μm, increasing the efficiency of inter-channel signal transmission.

This approach to fluidic actuation permits simultaneous pulses in multiple channels, is applicable for any lab seeking to employ microfluidics, and does not require expensive pumping systems. Like neurons, glial cells can be guided into adjacent microchannel compartments. Figure 7A,B show that individual glial cells cultured in E-PDMS microfluidics develop glial extensions that migrate along channel corners to extend through 3 µm interconnects. Larger ramified glial cells do not prefer channel corners (Figure 7C) but appear to avoid them; however, most glia show affinity for the glass-to-sidewall channel interface (Figure 7D,E). Similar observations of corner affinity, or avoidance, have been noted for other cell types in microfluidics [22,44]. Glial protrusions possess sensory capabilities for detecting changes in the extracellular concentrations of small signaling molecules (e.g., ATP, glutamate). Glial cells exhibit the spontaneous or evoked fluctuations of internal Ca^2+^ stores that can induce propagating signals that pass through nervous tissues and cell cultures [45,46,47]. Potential roles of Ca^2+^ waves include contributions to vascular regulation, metabolic processes, synaptic modulation, and mechanisms of axonal guidance [48,49,50]. Ca^2+^ transients in glial cells exhibit characteristic temporal signature forms and periodicities [51,52,53].

**Figure 8:**
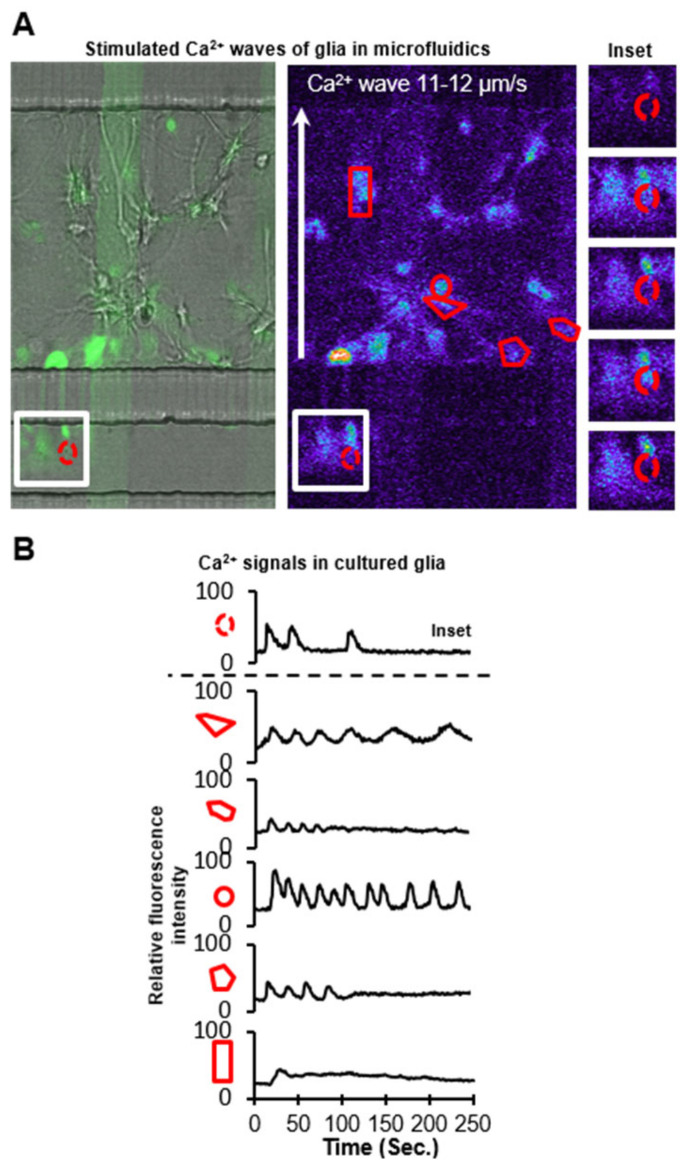
Ca^2+^ signals of glial networks in E-PDMS microfluidics. (**A**) Primary glial cells proliferate and extend branches into adjacent microchannels through interconnects. All channels were loaded with Ca^2+^ indicator (Fluo-4 AM in PBS). Glial cell branches were stimulated via 10 µM ATP infusion (**left** to **right**) through stimulation channel (**bottom**). A combined brightfield and fluorescence image is shown (**left**) alongside the respective fluorescence intensity glow-scale image (**right**); FITC-PLL lines (green) on glass are reference markers. Segmented images of inset (**right**) demonstrate repeated Ca^2+^ fluctuations. (**B**) Fluorescence profiles of Ca^2+^ responses from respective ROI demonstrate the range of characteristic Ca^2+^ responses for cultured astrocytes. Glia cultured in microdevices exhibit a typical range of Ca^2+^ fluctuations that vary in frequency, amplitude, and duration.

Figure 8A shows a population of glial cells stimulated through a subcellular plug of ATP (10 μM) focally administered to a compartmentalized cell. Figure 8B shows that, within the population, some glial cells have a brief, robust cyclic or acyclic response, while other cells continue to oscillate beyond the 4 min observation time. Previous studies have typified Ca^2+^ responses with high-throughput analyses; signals of glia in E-PDMS microfluidics conform to categorical morphologies of spikes, bursts, cyclic responses, or sustained Ca^2+^ elevations [51]. The rate of Ca^2+^ wave propagation (11–12 µm/s) for glia cultured in our E-PDMS microfluidic samples are within the range of wave propagations observed in dispersed cultures and brain slices (6–27 µm/s) [54]. The process of extracting unpolymerized oligomers and binding E-PDMS to the glass through autoclaving both removes harmful oligomers that can accumulate in cells [22,24] and provides strong normal adhesion forces that allow for cell signaling studies without E- PDMS delamination. With the ability to develop and stimulate compartmentalized glial cultures without biasing the remaining culture to stimulating chemical cues, we cultured glial populations in the central channel between two parallel microfluidic stimulation channels.

**Figure 9:**
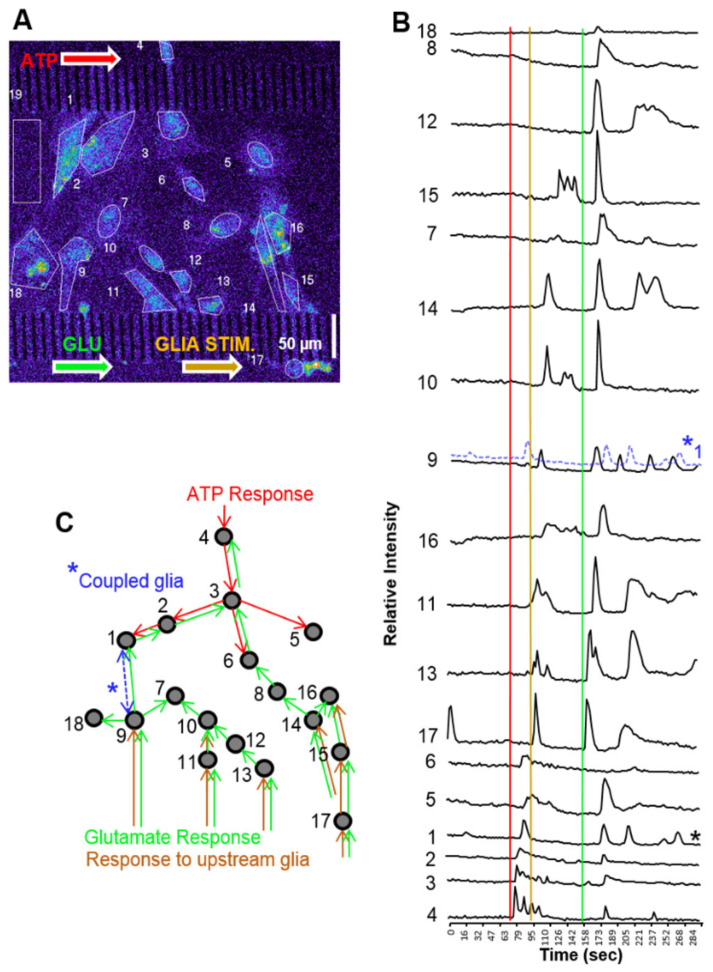
Glial network activation through multipoint stimulation. (**A**) Glial cells were cultured in the central channel of a microfluidic platform for stimulation at opposite sides of the culture for observing network activity. First, 20 µM ATP was introduced, then 50 µM glutamate was inserted into the bottom channel. From two chemical stimulations, three network activations occur. (**B**) Background (ROI#19)-subtracted fluorescence intensity profiles of each defined RIO ordered in chronological activation. Temporally coupled glial (*) signals from cellular ROI 1 and 9 suggest the two cells are electrically coupled. Glial cell 1 signal (*1) is superimposed with coupled glia 9 for comparison. (**C**) Schematic summary of Ca^2+^ signal propagation through the glial population.

Glial cells develop in the central channel and expand in clusters to extend processes into adjacent channels throughout the length of the microfluidic platform (Figure 9). ATP (20 µM) was administered before glutamate (50 µM) in quick succession (73 s between applications) (Figure 9A). Although two inputs were applied, three distinct population responses were observed (Figure 9A,B). First, ATP created a local response on one side of the population. A second stimulation locally activated the opposite portion of the population, followed by a third robust response on the same side as the second response. The third stimulation induced the activation of the entire population, reminiscent of a microburst (Figure 9B). While the source of the additional Ca^2+^ response is not known, the most plausible explanation would be that the ATP plug activated an upstream glial population and that glial processes extending into the second stimulation channel may have released a chemical messenger into the channel prior to the introduction of the glutamate application. Through laminar flow, the chemical messenger could be carried down the channel to activate downstream glial populations. Figure 9C schematic summarizes the sequence of cellular “regions of interest” activated and displayed in Figure 9B.

Glial cells are known to signal both through gap junctions and vesicular release [48,55]. Gap junctions permit local signal transfer to directly connected cells, whereas gliotransmitter release can influence cells not connected through gap junctions [56]. We demonstrate the ability to culture glial cells in E-PDMS microfluidic devices for cell signaling investigations. A handful of studies co-culture glial cells with neurons in microfluidics for studying neuro-glial interactions [57,58,59,60,61,62]. To our knowledge, no investigations specifically study glial population signaling interactions in microfluidic devices.

Microfluidic environments surpass cell culture dishes in their ability to exert spatiotemporal control of the microenvironment. Glial cells stimulated in a dish can release gliotransmitters that diffuse from sites of stimulation to neighboring cells. Culture-dish perfusion chambers allow for continuous fluidic exchange that can rapidly wash stimuli away; however, the entire culture is exposed to flow conditions that carry the stimuli across the remaining population or dilute chemical messengers released locally. Microfluidic environments overcome many of the limitations of dish cultures, perfusion chambers, or in vivo, while enabling signaling studies not previously achievable.

## 4. Conclusions

This work advances the fabrication and implementation of microfluidics without conventional plasma-generating systems that are typically required for assembling microfluidic systems, particularly for biological applications and cell signaling studies. The assembly process defined here is compliant with biological cell culture preparations and infrastructure (autoclave and solvent hood) available in biological departments. We show that elevated humidity and temperature can be used to induce high-strength bonds between E-PDMS and glass to permit highly biocompatible microfluidics for maintaining fluidic fidelity during growth and stimulation studies. An advantage of this annealing process is that humidified cell culture conditions (37 °C) favor the bonding process of E-PDMS to glass rather than counteract it.

This simple, effective fabrication method will expand the range of possibilities for process miniaturization and sample manipulation in tightly bound, biocompatible, PDMS-based microfluidics. Solvent extraction or autoclaving alone improves material biocompatibility. Combining these easy processes in sequence retains material biocompatibility while increasing material adhesion, thus improving the versatility of applications of E-PDMS for microfluidic platforms. It is yet to be determined how E-PDMS microfluidics will be advantageous for chemical synthesis, material interactions, flexible electronics, or PDMS surface modifications, but the implications from PDMS deformation from oligomer translocation may prove valuable.

Oligomer translocation between PDMS types is evident through material deformation when oligomer-free, E-PDMS is placed in contact with oligomer-containing PDMS and subjected to elevated temperatures. This provides a possible approach for optimizing curing agent and pre-polymer ratios and curing conditions to minimize oligomer translocation through, and out of, PDMS.

Optimal cell signaling results when environmental confounds are eliminated. E-PDMS microfluidics are devoid of free oligomers, which are known to accumulate in cells and modify gene transcription. E-PDMS microfluidic platforms are advantageous for a wide range of signaling studies for monotypic cell cultures or co-cultures.

## Data Availability

Not applicable.
